# Next generation sequencing and molecular imaging identify EGFR mutation and amplification in a glioblastoma multiforme patient treated with an EGFR inhibitor: a case report

**DOI:** 10.18632/oncotarget.18148

**Published:** 2017-05-24

**Authors:** Ke Zhou, Hui Yao, Xuewen Zhang, Jiangang Liu, Zhenyu Qi, Xueshun Xie, Xiaoting Xu, Youxin Zhou, Zhengquan Yu, Zhong Wang, Yanjun Che, Yulun Huang

**Affiliations:** ^1^ Department of Neurosurgery and Brain and Nerve Research Laboratory, The First Affiliated Hospital of Soochow University, Suzhou, China; ^2^ Department of Radiotherapy, The First Affiliated Hospital of Soochow University, Suzhou, China; ^3^ Department of Neurosurgery, The Jingjiang People's Hospital, Taizhou, China

**Keywords:** EGFR mutation, glioblastoma, molecular image, erlotinib, next-generation sequencing

## Abstract

Epidermal growth factor receptor (EGFR) mutations and amplifications are frequently reported in glioblastoma multiforme (GBM) patients. In this case report, we utilize next-generation sequencing (NGS) and EGFR molecular imaging to investigate intratumoral heterogeneity in a male patient presenting with GBM. Further, we describe the patient's clinical course as well as outcomes of targeted EGFR therapy with erlotinib, an EGFR tyrosine kinase inhibitor (TKI). NGS demonstrated the presence of an EGFR mutation and amplification in our patient. Molecular imaging revealed a heterogeneous expression pattern of EGFR in the frontal and temporal lobes. This patient briefly responded to erlotinib therapy. However, the patient relapsed and died from progressive neurological deterioration. Partial response and acquired secondary resistance may be attributed to intratumoral heterogeneity. Combination of NGS and EGFR molecular imaging may be helpful in understanding intratumoral molecular heterogeneity and may aid in developing individualized GBM treatments, thereby improving outcomes.

## INTRODUCTION

Glioblastoma multiforme (GBM) is a highly malignant tumor of the central nervous system. The overall median survival time of GBM is approximately 12-15 months [1, 2]. Currently, GBM is primarily treated with surgical resection, followed by a combination of radiotherapy and chemotherapy.

Epidermal growth factor receptor (EGFR) is a transmembrane receptor tyrosine kinase protein that has been associated with several human malignancies. EGFR associated mutations, amplification or overexpression are observed in approximately 50 % of glioblastoma patients [3]. EGFR amplification has been proposed as a marker of poor prognosis [4], however, EGFR-targeted therapy can be a promising treatment for GBM patients.

Erlotinib (Tarceva) is an oral EGFR tyrosine kinase inhibitor (TKI) that has been extensively clinically validated. However, patients’ response rate ranges from 10 to 20 % [5–7]. Phase II clinical trials revealed that the single-agent activity of erlotinib was marginally beneficial for GBM patients following radiotherapy [5]. Moreover, erlotinib demonstrated insufficient single-agent activity over standard therapies in GBM patients [8]. Therefore, identifying markers that can predict the outcome of erlotinib therapy may be beneficial for GBM patients and might uncover factors that improve treatment sensitivity. *PTEN* is a tumor suppressor gene that is commonly mutated in glioblastoma [9]. The expression of amplified and aberrant EGFR combined with the expression of wildtype PTEN were important predictors for the sensitivity towards EGFR kinase inhibition in glioblastoma xenografts [10]. In GBM patients, co-expression of EGFRvIII and PTEN was significantly associated with a favorable clinical response [11]. Moreover, GBM patients with higher levels of EGFR expression and lower levels of phosphorylated PKB/Akt demonstrated improved sensitivity to erlotinib treatment [12]. Nevertheless, to date, the molecular characteristics of GBM subpopulations of patients that demonstrate higher responses towards TKIs have not been fully elucidated [13]. Here we describe a GBM patient who had a short-term response to TKI. We used next generation sequencing (NGS) and molecular imaging to investigate the presence of EGFR mutations.

## CASE PRESENTATION

**Figure 1:**
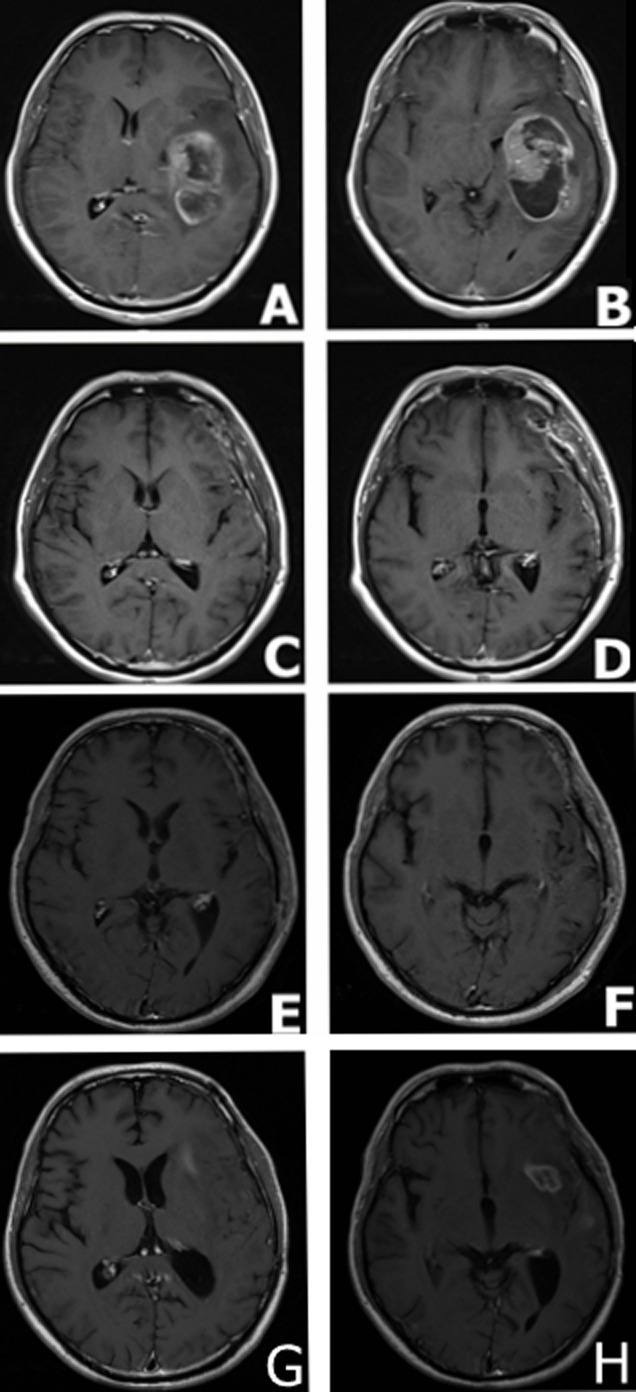
MRI findings in a male patient presented with glioblastoma multiforme **A**. and **B**. MRI scans at the disease onset demonstrating a large mass in the left temporal parietal area with marked surrounding edema and a shift of the midline structures to the left side. **C**. and **D**. MRI scans captured at one month following surgical resection; MRI demonstrating gross total resection. **E**. and **F**. MRI scans at 5 months following surgical resection, standard radiation and concomitant chemotherapy demonstrated the absence of tumor relapse. **G**. and **H**. MRI scans at 7 months following surgical resection: MRI demonstrated tumor relapse.

**Figure 2:**
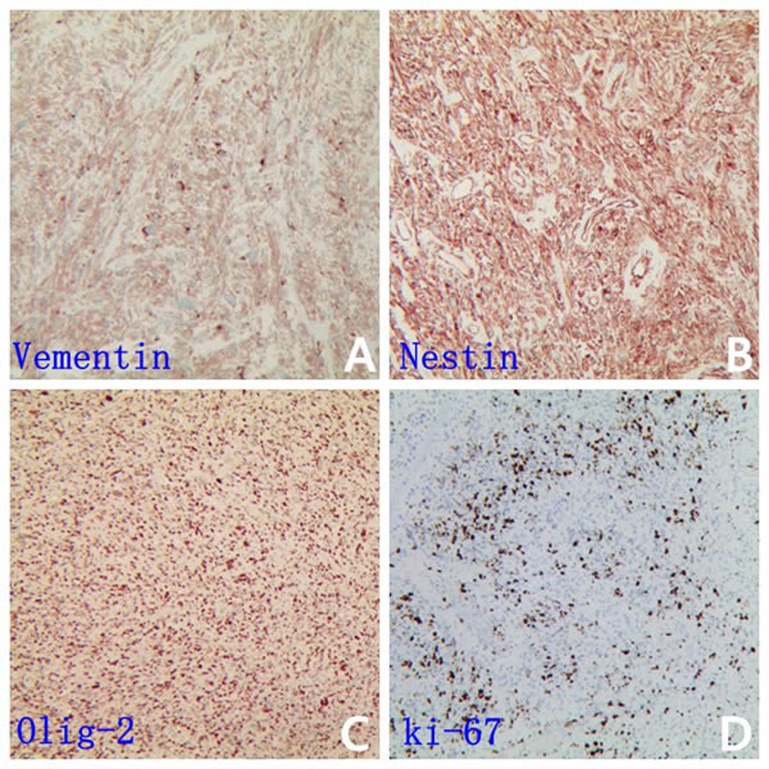
GBM biomarkers is immunopositive in patient specimen Representative IHC images are shown for vimentin **A.**, nestin **B.**, Olig-2 **C.** and the ki67 **D.** (which labeling index was 70 %)

A 31-year-old male was admitted to the First Affiliated Hospital of Soochow University, China complaining from headache, vomiting, and mild left hemiparesis. Magnetic resonance imaging (MRI) revealed a large abnormal mass in the left temporal parietal area with marked edema and a shift of the midline structures to the left side (Figure 1A and 1B). The patient was diagnosed with glioblastoma and underwent gross total resection in February 2015. The tumor tissue was preserved for immunohistochemical study which revealed immunopositive reactions against the GBM biomarkers GFAP, CD56, vimentin, nestin and Olig-2 (Figure 2A, 2B and 2C). Further, the ki67 labeling index was 70 % (Figure 2D). The gross total resection of the GBM was confirmed by a follow up MRI performed at one month post-operatively (Figure 1C and 1D). The patient received treatment according to Stuup et al [14] regimen of standard radiation and concomitant temozolomide chemotherapy. After radiation, MRI verified that the patient had not relapsed (Figure 1E and 1F). His therapeutic regimen was composed of six adjuvant temozolomide cycles (first cycle was 150 mg/m^2^/day, the remainder of the cycles were 200 mg/m^2^/day) for five days every 28 days. By September 2015, our patient successfully finished five cycles of adjuvant temozolomide, however, routine follow up MRI revealed the relapse of the GBM prior to commencement of the sixth temozolomide cycle (Figure 1G and 1H).

Subsequently, we performed NGS by using tissues obtained at diagnosis in order to investigate the molecular characteristics of the temozolomide resistant GBM and to identity new therapeutic strategies. Total DNA was extracted from tumor paraffin sections using the GeneRead^TM^ DNA FFPE Kit (Qiagen, Germany), according to the manufacturer's protocol. Genomic DNA was fragmented into fragments ranging from 300-350 bp using a focused-ultrasonoscope (Covaris M220, USA). Agilent SureSelect XT reagents were used to prepare sequencing libraries according to the manufacturer's protocol. Hybrid capture was conducted using Agilent SureSelectXT Human All Exon V6. After PCR amplification, the library was created using Bioanalyzer 2100 (Agilent, USA) and AriaMx Real-Time PCR system (Agilent, USA). The library was sequenced on Illumina HiSeq4000 Analyzers (Illumina, USA) for 151 cycles to generate 150 bp paired-end reads. Image analysis and base calling were performed using the Illumina Pipeline. Sequencing depth was 500-8000X. NGS revealed the following results: EFGR amplification 14 times, EGFR p.A289V mutation, pP772 delinsPP mutation and FLNA point mutation (Table 1). Further, data analysis identified EGFR as a driver gene.

**Table 1:** Mutation analysis of glioblastoma multiforme patient tumor

Gene	Variation type	Nucleotide variation	Amino acid variations	Sequencing depth	Mutation frequency
EGFR	point mutation	c.C866T	p.A289V	7941	95%
EGFR	insertion mutation	c.2318_2319insACC	p.P773delinsPP	7932	9.50%
FLNA	point mutation	c.G3718A	p.V1240M	250	20.80%

EGFR gene amplification 14 times

**Figure 3:**
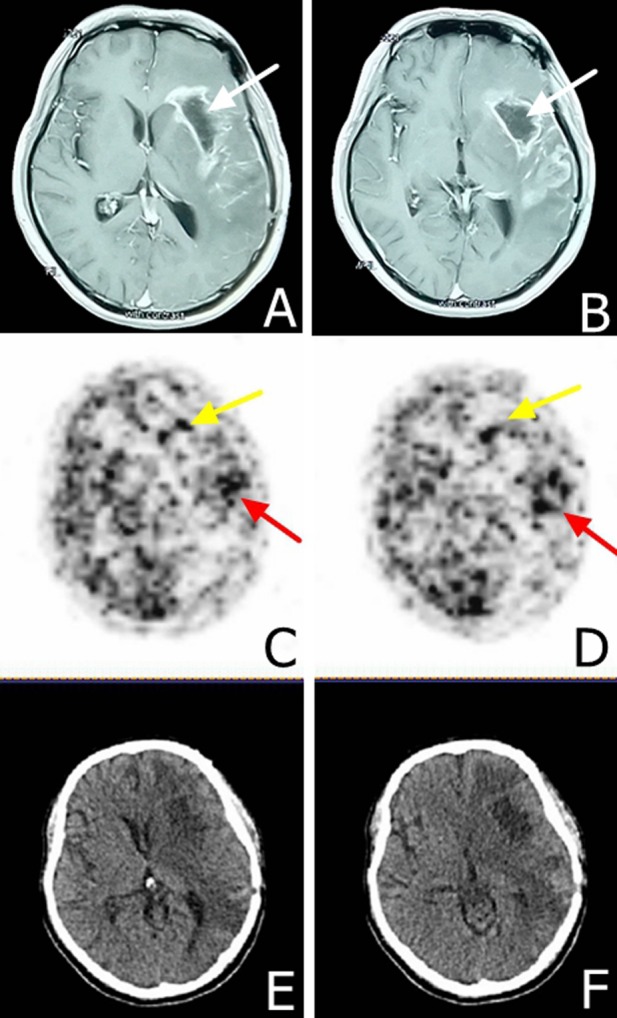
MRI and EGFR-18F-FDG and PET/CT scans in a male patient presented with glioblastoma multiforme at eight months following surgical resection **A**. and **B**. MRI scans demonstrating the tumor relapse at eight months following surgical resection. The white arrow shows the tumor hollowing. **C**. and **D**. EGFR-18F-FDG, PET/CT scans demonstrate heterogeneous tumor characteristics at 48 hours post-tracer injection, yellow arrows demonstrate area with lower staining intensity in the frontal lobe and the red arrow demonstrates a higher staining intensity in the temporal lobe. **E**. and **F**. CT scan indicating tumor hollowing and significant edema.

Next, we evaluated EGFR expression in the patient's brain by molecular imaging at eight months post-operatively. EGFR antibody-18-FDG was used as a tracer agent. We injected the EGFR antibody-18-FDG and performed a PET/CT scan at 24, 48, 72 hours post-injection. We observed significant tracer uptake by the tumor at 24, 48, and 72 hours. The standardized uptake value (SUVmax) of tumor/ non-tumor was 6.4, 10, and 8.2 for the 24, 48 and 72 hours, respectively (Figure 3 and data not shown). Furthermore, the temporal lobe of the brain showed a strong positive signal while the frontal lobe showed a weak positive signal.

**Figure 4:**
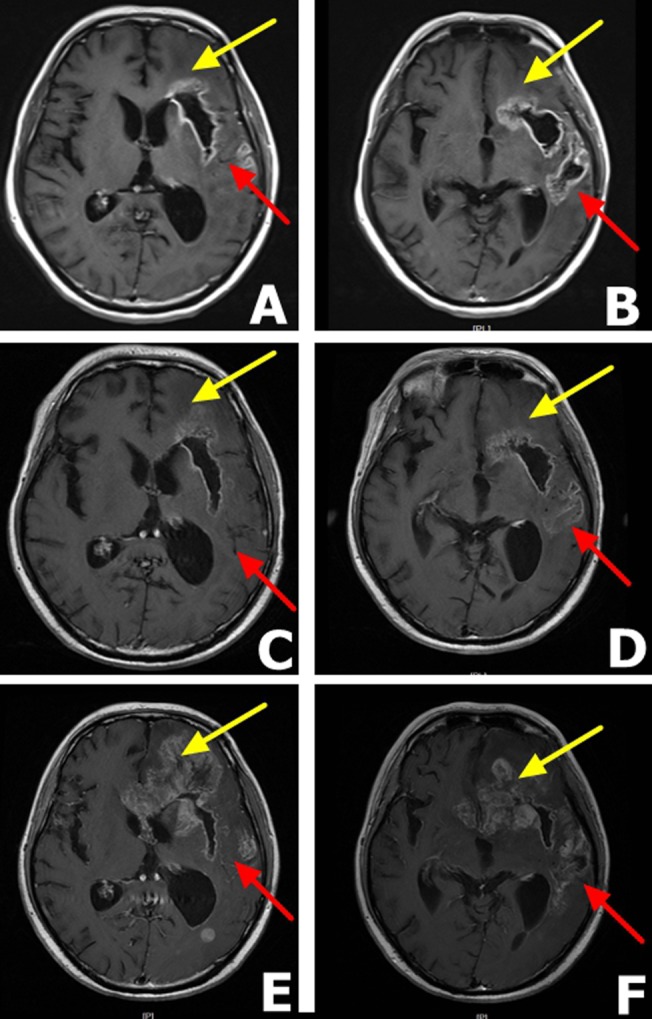
MRI findings in a male patient presenting with glioblastoma multiforme after erlotinib 150 mg therapy A. and B. MRI scans after 14 days of daily erlotinib 150 mg therapy The scans reveal tumor hollowing, lower grade edema and reduced compression of the lateral ventricles. **C**. and **D**. Three months after erlotinib 150 mg therapy, MRI scans shows that the tumor stabilized. Yellow arrow shows a stable frontal lobe while the red arrow show partial response in the temporal lobe. **E**. and **F**. Five months after erlotinib 150 mg therapy. MRI scans demonstrate the tumor relapsed for the second time with distant metastasis and the patient died two months later. The yellow arrow shows obvious metastasis in the frontal lobe and red arrow shows a stable temporal lobe.

Given the above-mentioned results, we decided to administer TRI therapy with erlotinib 150 mg/day starting from November 2015. The patient showed improvement over four days; he showed no dysarthria and no gait abnormalities. MRI scan revealed tumor hollowing and improvement in edema at 14 days post-erlotinib treatment (Figure 4A and 4B). Follow up MRI scans were carried out monthly. In the first three months, GBM tumor was improved with less observed edema and reduced compression of the lateral ventricles (Figure 4C and 4D). However, at the fourth month, our patient should progressive neurological deterioration and the MRI scan revealed the recurrence of GBM. In the frontal lobe, the tumor progressed rapidly, extended to corpus callosum and to the right lobe (Figure 4E and 4F). The patient died after three months from the secondary progression in May 2016. Overall survival time was 15 months for the patient. Progression of the patient's illness and the therapeutic regimen are summarized in Table 2

**Table 2:** Progression of the patient's illness and the therapeutic regimen

Time	Patient history and treatment regimen
Feb, 2015	Headache, vomiting, and mild left hemiparesis. The patient was diagnosed with glioblastoma and underwent gross total resection.
March/April, 2015	Standard radiation and concomitant temozolomide chemotherapy.
May, 2015-Sept, 2015	Adjuvant temozolomide 5 cycles (150mg/day in first cycle, 200mg/day in other cycle)
Sept, 2015	The relapse of the GBM
Oct, 2015	Next generation sequencing demonstrates EGFR mutation
Oct, 2015	Perform a PET/CT by EGFR antibody-18-FDG shows non-uniform EGFR positive reaction
Nov, 2015	Oral administration of erlotinib 150 mg/day
Nov, 2015-Feb, 2016	SD (less edema and reduced compression of the lateral ventricles)
March, 2016	Recurrence of GBM
May,2016	Patient death

GBM: Glioblastoma multiforme SD: Stable Disease

The patient provided informed written consent for the publication of this case report and all accompanying images.

## DISCUSSION

GMB is often associated with the mutation and amplification of the *EGFR*. Further, *EGFR p.A289V* mutation is most common mutation associated with GBM [15]. However, the presence of multiple or heterogeneous types of mutations is a hallmark of GBM. Using deep sequencing, Kumar et al. previously identified spatial heterogeneity in *Tp53*, *EGFR*, and *PDGFRA* genes in glial tumors [16]. Furthermore, Francis et al., developed a novel approach to identify distinct tumor subpopulations from the bulk tumor using single-cell whole-genome sequencing allowing them to infer the subclonal architecture. Detailed mutational analysis of tumors will aide in identifying mechanisms underpinning drug resistance which may ultimately improve the effectiveness of personalized cancer treatment [17]. The overexpression of EGFR is a predictive biomarker for response to different therapeutic regimens. Indeed, molecular imaging is a non-invasive technique that enables the detection of EGFR overexpression. Therefore, molecular imaging can aid in identifying the patient population with positive EGFR expression and hence can benefit from targeted TKI-based therapies.

In this case report, we described the molecular imaging and NGS in a male patient presenting with GBM. Results of EGFR-18F-FDG PET/CT scan demonstrated heterogeneous expression of EFGR protein in our male patient. We observed heterogeneity in the intensity of EGFR staining across the brain. The temporal lobe of the brain showed a strong positive signal while the frontal lobe showed a weakly positive signal. It is plausible to speculate that the heterogeneity of EFGR expression may result in variable efficacy of TKI therapy [18]. Further, NGS revealed the presence of EFGR amplification and the EGFR mutations p.A289V and pP772 delinsPPl, as well as the FLNA point mutation p.V120M. In the first three months of oral erlotinib treatment, the patient's symptoms improved. However, the efficacy of single erlotinib therapy declined with a relapse in the patient's condition. In the frontal lobe, the GBM expanded, extending to the corpus callosum and to the contralateral lobe. As discussed earlier, the frontal lobe showed lower EGFR activity which may have affected sensitivity towards erlotinib, leading to secondary resistance.

The existence of intratumoral heterogeneity has been demonstrated to possess prognostic implications and may affect the sensitivity towards chemotherapy [19]. Further, intratumorral diversity represented a probable cause for anti-EGFR therapeutic secondary resistance. Due to trimming of tissues obtained from surgical resection, tumor samples do not reflect the molecular representation of the whole tumor [19]. Moreover, Wei et al. previously demonstrated that tumors evolve in response to targeted therapies through the classical Darwinian selection and cellular adaptations at a variety of levels [18]. In this study, we demonstrated that the combination of NGS and molecule imaging may be instrumental in studying the complex molecular biology and intratumoral molecular heterogeneity. Further, we observed that single target inactivation was not sufficient to block downstream oncogenic signaling. Our male patient responded briefly to erlotinib before acquiring secondary resistance. It has been previously demonstrated that combining erlotinib with histone deacetylase inhibitors (HDACi), significantly inhibit the proliferation of erlotinib-resistant GBM cells and partially restore their sensitivity to erlotinib [20]. Moreover, targeting MET, a hepatocyte growth factor receptor, in GBM cases with EGFR amplification may delay the acquired secondary resistance that can develop during erlotinib treatment [21]. Taken together, the combination of erlotinib with other drugs can potentially prevent TKI secondary resistance. Therefore, we recommend combining two or more target drugs which may aide in prolonging survival in GBM patients [18].

In conclusion, NGS and molecular imaging can provide crucial information about the molecular composition of tumors which will help in developing individualized targeted therapy.
